# Comparison of Bone and Renal Effects In HIV-infected Adults Switching to Abacavir or Tenofovir Based Therapy in a Randomized Trial

**DOI:** 10.1371/journal.pone.0032445

**Published:** 2012-03-29

**Authors:** Thomas A. Rasmussen, Danny Jensen, Martin Tolstrup, Ulla S. Nielsen, Erland J. Erlandsen, Henrik Birn, Lars Østergaard, Bente L. Langdahl, Alex L. Laursen

**Affiliations:** 1 Department of Infectious Diseases, Aarhus University Hospital, Aarhus, Denmark; 2 Department of Nephrology, Aarhus University Hospital and Institute of Biomedicine, Aarhus University, Aarhus, Denmark; 3 Department of Clinical Biochemistry, Viborg Regional Hospital, Viborg, Denmark; 4 Department of Endocrinology and Internal Medicine, Aarhus University Hospital, Aarhus, Denmark; University of New South Wales, Australia

## Abstract

**Introduction:**

Our objective was to compare the bone and renal effects among HIV-infected patients randomized to abacavir or tenofovir-based combination anti-retroviral therapy.

**Methods:**

In an open-label randomized trial, HIV-infected patients were randomized to switch from zidovudine/lamivudine (AZT/3TC) to abacavir/lamivudine (ABC/3TC) or tenofovir/emtricitabine (TDF/FTC). We measured bone mass density (BMD) and bone turnover biomarkers (osteocalcin, osteocalcin, procollagen type 1 N-terminal propeptide (P1NP), alkaline phosphatase, type I collagen cross-linked C-telopeptide (CTx), and osteoprotegerin). We assessed renal function by estimated creatinine clearance, plasma cystatin C, and urinary levels of creatinine, albumin, cystatin C, and neutrophil gelatinase-associated lipocalin (NGAL). The changes from baseline in BMD and renal and bone biomarkers were compared across study arms.

**Results:**

Of 40 included patients, 35 completed 48 weeks of randomized therapy and follow up. BMD was measured in 33, 26, and 27 patients at baseline, week 24, and week 48, respectively. In TDF/FTC-treated patients we observed significant reductions from baseline in hip and lumbar spine BMD at week 24 (−1.8% and −2.5%) and week 48 (−2.1% and −2.1%), whereas BMD was stable in patients in the ABC/3TC arm. The changes from baseline in BMD were significantly different between study arms. All bone turnover biomarkers except osteoprotegerin increased in the TDF/FTC arm compared with the ABC/3TC arm, but early changes did not predict subsequent loss of BMD. Renal function parameters were similar between study arms although a small increase in NGAL was detected among TDF-treated patients.

**Conclusion:**

Switching to TDF/FTC-based therapy led to decreases in BMD and increases in bone turnover markers compared with ABC/3TC-based treatment. No major difference in renal function was observed.

**Trial registration:**

Clinicaltrials.gov NCT00647244

## Introduction

Highly active anti-retroviral therapy (HAART) has substantially reduced mortality and morbidity from human immunodeficiency virus (HIV) infection. With effective HAART HIV-infected patients have a life expectancy approaching that of the general population [1], but are faced with life-long treatment. Understanding the long-term consequences, including bone and renal effects, of HIV-infection and anti-retroviral exposure is therefore of increasing importance.

At the initiation of this study fixed-dose combinations of tenofovir (TDF)/emtricitabine (FTC) or abacavir (ABC)/lamivudine (3TC) were recommended for initial treatment of HIV infection [2], [3]; TDF/FTC was later recommended over ABC/3TC in some guidelines [4].

Previous studies have indicated an increased prevalence of osteopenia and osteoporosis [5], as well as increased rates of fractures [6], among HIV-infected individuals compared to HIV-uninfected controls, but the relative contribution to this of HIV-infection, anti-retroviral therapy exposure, and traditional risk factors is still not completely resolved. Results from several randomized clinical trials among anti-retroviral naïve patients have shown that initiation of HAART is associated with a decrease in bone mineral density (BMD) of 2%–6% at both the hip and spine that occurs within the first year of treatment with stabilization thereafter [7]–[13]. Of these, two trials directly compared TDF/FTC with ABC/3TC and both found greater decreases in BMD with TDF/FTC-based treatment than with ABC/3TC-based treatment [11], [13]. To date, only one trial has reported on BMD among virologically suppressed HIV-infected patients switching to TDF/FTC or ABC/3TC-based treatment. In this study, patients randomized to TDF/FTC experienced decreases in BMD compared with ABC/3TC as measured by dual energy x-ray absorptiometry (DXA) [14].

Case reports of renal dysfunction associated with TDF therapy [15], [16] raised concerns of a nephrotoxic effect with this compound, but the incidence of adverse renal effects were low in initial randomized trials and comparable to thymidine analogues [7], [17]. In a recent meta-analytic review of prospective studies comparing TDF-containing with non-TDF containing HAART-regimes a small but statistical significant loss of renal function was associated with TDF use [18]. Included herein are 3 studies comparing TDF with ABC with mixed results on the renal endpoints [19]–[21]. In addition, there was no difference in estimated glomerular filtration rate (GFR) between patients randomized to ABC- or TDF-based therapy in the STEAL [14] or ASSERT-study [22], but increases in markers of proximal tubule dysfunction were observed in the TDF-arm of the latter. Some of the discrepancy is likely related to differences in selection criteria for study participants with regard to renal function.

The Efficacy and Safety of Switching from Zidovudine to Tenofovir or Abacavir in HIV-infected Patients (SWAP) study was a randomized trial comparing switching from zidovudine (AZT)/3TC to ABC/3TC or TDF/FTC. We have previously published a report on cardiovascular biomarkers from this trial [23] and here provide the main results from the trial. The aims of this study were to compare the effects of abacavir and tenofovir on renal function and bone mass density, including exploratory analyses of several biomarkers of bone turnover and renal dysfunction.

## Methods

The protocol for this trial and supporting CONSORT checklist are available as supporting information; see Checklist S1 and Protocol S1.

### Study participants

The SWAP study was an open-label, parallel-group, randomized clinical trial assessing the safety and efficacy of switching from AZT to ABC- or TDF-based therapy. The study design and patient group have been described previously [23]. In brief, eligible patients had documented HIV-infection and, for at least 3 months, had been treated with HAART comprising AZT and had undetectable plasma HIV-RNA. The pre-specified primary endpoints were renal function assessed by cystatin C (cysC), creatinine clearance (CrCl) and markers of tubule function; bone mass density and bone metabolism assessed by DXA and bone turnover markers; lipodystrophy and body composition assessed by DXA and patient questionnaire; and insulin sensitivity. A sample size of 90 participants was estimated as being required to detect differences in the renal tubular function at a 5% significance level based on existing limited knowledge on the frequency of impaired tubular function with tenofovir and abacavir based therapy. Patients who had previously used ABC or TDF, had diabetes mellitus, untreated hypertension, renal disease, osteoporosis or who were positive for the HLAB5701 allele upon screening were excluded. Informed written consent was obtained before enrolment. At baseline, patients were stratified after use of protease inhibitors and randomized 1∶1 to switch from AZT to ABC 600 mg or TDF 300 mg daily using sequentially numbered envelopes generated by the Aarhus University Hospital Pharmacy. The treating physician assigned patients to intervention. If lamivudine (3TC) or emtricitabine (FTC) was given as part of HAART at inclusion, patients were randomized to a fixed-dose treatment with ABC/3TC 600/300 mg or TDF/FTC 300/200 mg daily. The study took place at the Department of Infectious Diseases, Aarhus University Hospital, Skejby, Denmark. Patients were followed up at 4, 8, 12, 24, and 48 weeks after randomization. A pre-planned follow up at 96 weeks was not performed because the study was discontinued in November 2009 before reaching the intended number of study participants. For this reason, and lack of funding, final analyses were restricted to bone, renal, and cardiovascular investigations. No interim analyses were planned and none was performed before recruitment was stopped. The study was approved by the Regional Research Ethics Committee, Central Denmark Region (Journal number: M-20070189; Eudract. number: 2007-004372-39) and conducted in accordance with *Good Clinical Practice*. Anthropometric information and data on age, sex, smoking habits, blood pressure, CD4+ cell counts, viral load, and duration of HIV-infection and HAART exposure were retrieved from the study database or patient records.

### BMD and biomarkers of bone turnover

DXA scans (DXA machine Hologic Discovery A (S/N 82962)) of the lumbar spine and hip were performed at baseline, week 24 and 48 at The Department of Endocrinology and Internal Medicine, Aarhus University Hospital, Denmark. Serum and plasma samples were collected at baseline and week 4, 12, 24, and 48. Samples were stored at −80°C until analyses. We measured 2 biomarkers of bone formation, osteocalcin (OC) and procollagen type 1 N-terminal propeptide (P1NP;) [24], [25] in plasma at baseline and week 4, 12, and 48 (both with a luminometric assay on Cobas 6000E, Hoffmann-La Roche; OC reference range 10–53 µg/L for women and 13–77 µg/L for men; P1NP reference range <62 µg/L for premenopausal women, <93 µg/L for postmenopausal women, and 20–76 µg/L for men). Plasma alkaline phosphatase levels were measured by a micro slide assay (Vitros FS 5.1, Ortho-clinical Diagnostics, reference range 35–105 U/L) at baseline and week 4, 12, 24, and 48. Plasma levels of type I collagen cross-linked C-telopeptide (CTx), a biomarker of bone resorption [24], [25], and osteoprotegerin (OPG) that is competitive inhibitor of osteoclast activation and differentiation [25]–[27], were measured at baseline and week 4, 12, and 48. CTx was measured using a luminometric assay on Elecsys 2010 (Hoffmann-La Roche) (reference range 0.04–0.87 µg/L for men <70 years, 0.04–0.87 µg/L for premenopausal women, and 0.03–0.83 µg/L for postmenopausal women) and OPG was assayed by a commercially available enzyme-linked immunosorbent assay (ELISA) (reference range 0.6–6.9 pmol/L) as described by the manufacturer (Biomedica Gruppe, Vienna, Austria). Baseline levels of 25-OH D2/D3 vitamin D in serum were determined using a mass spectrometry assay (reference range 50–160 nmol/L). Previous plasma measurements of interleukin-6 (IL-6) and high sensitive C-reactive protein (hs-CRP) [23] were used to assess concurrent inflammation at baseline. All biomarkers were batch-tested.

### Markers of renal dysfunction

Twenty-four hour urine collection was done at baseline and week 12, 24, and 48, but was incomplete or omitted in several patients at various time points. Instead, we measured plasma creatinine and phosphate levels at baseline and week 4, 8, 12, 24, and 48 (Vitros FS 5.1 Ortho-clinical Diagnostics) and used the Cockcroft-Gault formula to estimate creatinine clearance (eCrCl). Plasma cysC was measured at baseline and week 48. In addition, we determined urinary levels of creatinine, albumin, cysC, and neutrophil gelatinase-associated lipocalin (NGAL) among patients with urine samples from at least 2 time points and used the ratios of albumin, cysC, and NGAL levels relative to urine creatinine concentration to identify signs of renal dysfunction. The change in ratio from baseline to the second measurement was compared across study arms regardless of which time the latter was performed. Plasma and urinary cysC was determined by a particle enhanced nephelometric assay using the N Latex cysC assay on the Behring Nephelometer II (Siemens Healthcare Diagnostics Products GmbH, Marburg, Germany) as described previously [28]; reference level for plasma cysC is 0.51–1.02 mg/L [29]. Urinary NGAL concentration was determined using a commercial ELISA assay (Bioporto). Urinary albumin was assayed on the Behring Nephelometer II (Siemens Healthcare Diagnostics Products GmbH, Marburg, Germany) and urinary creatinine was assayed using the Roche Modular Analytics P automated clinical analyzer (Roche Diagnostics, Mannheim, Germany).

### Statistics

Baseline data were compared using χ^2^-test for categorical variables and two-sample t-test or Wilcoxon rank sum test for continuous variables. Percentage changes from baseline to week 24 and 48 in total hip and lumbar spine BMD were computed and compared within and between study arms using one- and two-sample t-tests, respectively. The changes from baseline to the various time points in levels of bone and renal biomarkers were compared between study arms using two-sample t-test or Wilcoxon rank sum test, as appropriate. Data distribution was assessed through visual inspection of frequency histograms and normal probability plots; logarithmic transformation was explored on skewed data to allow parametric hypothesis testing whenever possible. Linear regression was used to explore whether changes in bone turnover biomarkers at early time points (baseline to week 4 and 12, respectively) predicted subsequent reductions in BMD at week 48. Statistical analyses were per-protocol as pre-specified in the study protocol. *P*-values<0.05 were considered statistically significant. Stata 10 was used for statistical analyses.

## Results

### Study participants

**Figure 1:**
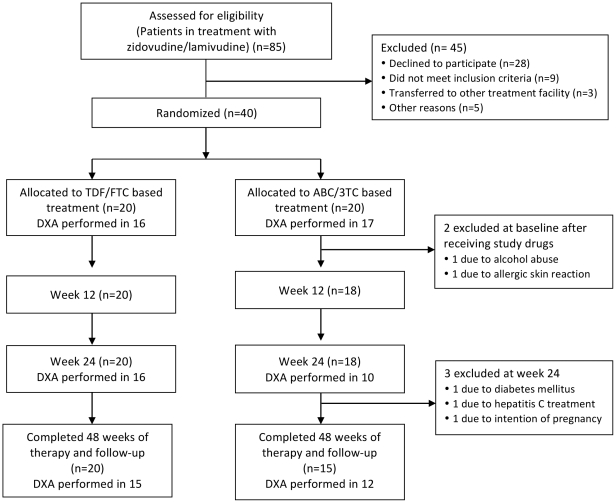
Flow chart of inclusion and follow up.

Details of study treatment and the patient group have been described previously [23]. Because of uncertainty about ABC as an appropriate first-line treatment option, patient inclusion was suspended in January 2009 and finally terminated in August 2009. By this time 40 patients had been enrolled (20 in each arm). Of these, 35 completed 48 weeks of therapy and follow-up (20 in the TDF/FTC arm and 15 in the ABC/3TC arm). The flow of patient inclusion and follow-up is illustrated in Fig. 1. Two patients in the ABC/3TC arm were excluded shortly after randomization and contribute only to baseline data. One patient was excluded because of an allergic skin reaction upon starting ABC therapy (the patient was tested negative for the HLAB5701 allele), and one patient was excluded because of alcohol abuse and poor adherence to study drugs. Three patients in the ABC/3TC arm were excluded at week 12 for the following reasons: initiated treatment for hepatitis C, intention of pregnancy, and a history of diabetes mellitus, respectively. The latter patient had a history of diabetes prior to randomization and was listed as a screening failure. All 3 patients contributed data up to and including week 12. The remaining 35 study subjects all completed 48 weeks of therapy and follow-up. One patient (TDF/FTC arm) did not provide the week-4 blood sample. One serious adverse event was reported during the study; this was a resolved episode of pyelonephritis in a patient with a history of recurrent urinary tract infections. Five grade 2 adverse advents were registered (4 in the ABC/3TC arm and 1 in the TDF/FTC arm), but none necessitated discontinuation of study drugs. No grade 3 or 4 adverse advents were reported.

None of the study participants received systemic corticosteroids, bisphosphonates, vitamin D, calcium or other therapies known to affect bone metabolism at enrolment or during the study. Baseline characteristics of the patients are shown in Table 1, including baseline levels of bone and renal biomarkers. Levels of P1NP were higher among patients in the ABC/3TC arm than the TDF/FTC arm (*P* = 0.040), but no other baseline parameter differed significantly between treatment groups, including levels of the inflammatory biomarkers, hs-CRP and IL-6.

**Table 1:** Baseline characteristics.

Parameter	TDF/FTC (n = 20)	ABC/3TC (n = 20)
**Anthropometric data**		
Mean age (95% CI), years	45.9 (41.8–49.9)	49.6 (44.0–55.1)
Male sex, n (%)	14 (70.0)	11 (55.0)
Active smokers, n (%)	9 (45.0)	6 (30.0)
Mean weight (95% CI), kg	74.9 (67.3–82.5)	73.2 (67.6–78.9)
Mean BMI (95% CI), kg/m^2^	25.0 (23.0–27.0)	25.2 (24.2–26.1)
**HIV-related data including HAART**		
Zidovudine/lamivudine at baseline, n (%)	20 (100)	20 (100)
PI at baseline, n (%)	6 (30.0)	6 (30.0)
Efavirenz at baseline, n (%)	9 (45.0)	11 (55.0)
Nevirapine at baseline, n (%)	5 (25.0)	3 (15.0)
Mean CD4+ cell count (95% CI), cells/mm^3^,	540 (444–636)	567 (447–687)
Median duration of HAART exposure (IQR), years	8.9 (4.2–10.8)	7.2 (4.5–10.4)
Median time since HIV-diagnosis (IQR), years	10.2 (7.4–14.9)	10.6 (8.0–11.7)
**Bone-related data**		
Mean lumbar spine BMD (95% CI), g/cm2	0.99 (0.93–1.04)	1.00 (0.95–1.05)
Mean hip BMD (95% CI), g/cm2	0.92 (0.86–0.98)	0.90 (0.84–0.97)
Median plasma osteocalcin (IQR), mikg/L	17.9 (15.3–22.5)	21.0 (17.5–27.2)
Median plasma P1NP (IQR), mikg/L	33.6 (29.8–48.2)	51.5 (33.8–62.6)
Median plasma CTx (IQR), ng/mL	0.25 (0.18–0.32)	0.34 (0.22–0.49)
Median plasma osteoprotegerin (IQR), pmol/L	3.4 (2.6–3.8)	3.1 (2.3–3.9)
Median plasma alkaline phosphatase (IQR), U/L	74.5 (65.5–90.5)	82.0 (68.5–99.5)
**Renal function-related data**		
Mean systolic blood pressure (95% CI), mmHg	135 (127–142)	133 (122–143)
Mean diastolic blood pressure (95% CI), mmHg	84 (80–89)	85 (79–90)
Mean fasting plasma glucose (95% CI), mmol/L	5.4 (5.2–5.5)	5.4 (5.2–5.6)
Median eCrCl a.m. Cockcroft-Gault (IQR), mL/min	130 (118–140)	120 (92–154)
Median urine NGAL/creatinine ratio (IQR)	0.42 (0.08–1.72)	0.28 (0.00–0.71)
Median urine cystatin C/creatinine ratio (IQR)	0.92 (0.55–1.54)	1.05 (0.54–1.15)
Median urine albumine/creatinine ratio (IQR)	0.39 (0.23–0.88)	0.47 (0.32–1.20)

BMI = body mass index; PI = protease inhibitor; HAART = highly active antiretroviral therapy; HIV = human immunodeficiency virus; BMD = bone mass density; P1NP = procollagen type 1 N-terminal propeptide; CTx = type I collagen cross-linked C-telopeptide; eCrCl = estimated creatinine clearance; NGAL = neutrophil gelatinase-associated lipocalin. Normally distributed data are presented as means with 95% confidence interval (95% CI) in parenthesis. Skewed data are presented as medians with interquartile range (IQR) in parenthesis.

### BMD and biomarkers of bone turnover

As DXA scan could not be conducted in all patients, changes from baseline in BMD was only evaluated for 26 (16 TDF/FTC arm and 10 ABC/3TC arm) and 27 (15 TDF/FTC arm and 12 ABC/3TC arm) patients at week 24 and 48, respectively. In 7 cases (4 TDF/FTC arm and 3 ABC/3TC arm) the week 4 measurements of OPG was omitted due to lack of plasma.

**Figure 2:**
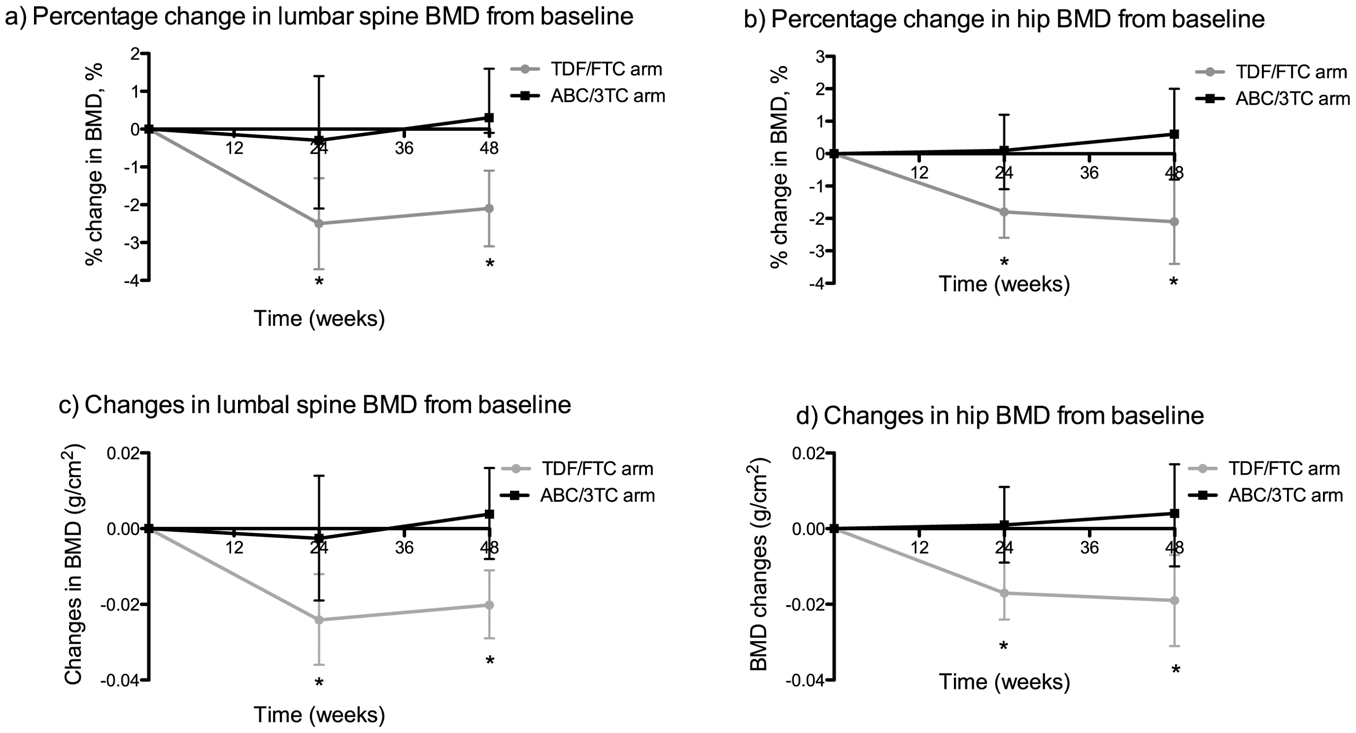
Changes from baseline in hip and lumbar spine BMD. Percentage and absolute mean changes from baseline in lumbar spine (a, c) and hip (b, d) bone mass density as measured by dual energy x-ray absorptiometry (DXA). BMD = bone mass density; TDF/FTC = tenofovir/emtricitabine; ABC/3TC = abacavir/lamivudine. Error bars show 95% confidence intervals.

We observed significant reductions from baseline in both hip and lumbar spine BMD at week 24 and 48 among TDF/FTC-treated patients, whereas BMD was stable in patients in the ABC/3TC arm. The mean percentage change at week 24 in hip and lumbar spine BMD was −1.8% (95% CI −2.6 to −1.1; *P*<0.001) and −2.5% (95% confidence interval (CI) −3.7 to −1.3; *P*<0.001) in the TDF/FTC arm compared to 0.1% (95% CI −1.1 to 1.2; *P* = 0.894) and −0.3% (95% CI −2.1 to 1.4; *P* = 0.671) in the ABC/3TC arm. At week 48 the mean percentage change in hip and lumbar spine BMD was −2.1% (95% CI −3.4 to −0.8; *P* = 0.004) and −2.1% (95% CI −3.1 to −1.1; *P*<0.001) in the TDF/FTC arm compared to 0.6% (95% CI −0.8 to 2.0; *P* = 0.400) and 0.3% (95% CI −0.9 to 1.6;*P* = 0.594) in the ABC/3TC arm. The percentage change from baseline in BMD was significantly different between study arms both at week 24 (*P* = 0.004 for hip and *P* = 0.033 for lumbar spine) and week 48 (*P* = 0.006 for hip and *P* = 0.003 for lumbar spine). Similar results were found for the absolute change in BMD in the two study arms (fig. 2).

**Figure 3:**
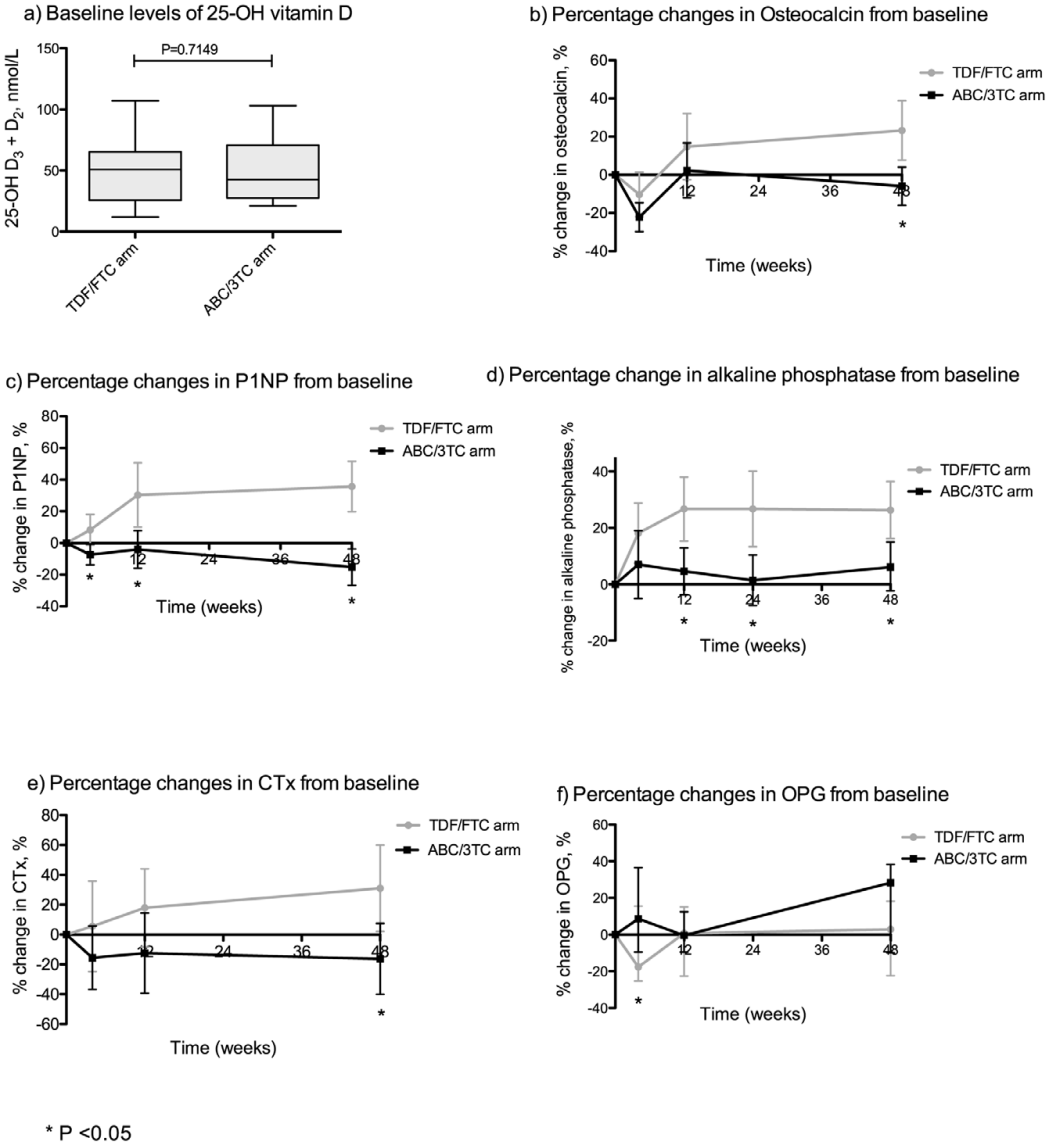
Changes from baseline in biomarkers of bone turnover. Baseline levels of 25-OH vitamin D (a) and percentage changes from baseline in biomarkers of bone turnover (b–f). P1NP = Procollagen type 1 N-terminal propeptide; CTx = Type I collagen cross-linked C-telopeptide (CTx); OPG = Osteoprotegerin; TDF/FTC = tenofovir/emtricitabine; ABC/3TC = abacavir/lamivudine. Mean values are depicted for normally distributed data and median values are depicted for skewed data. Accordingly, error bars show 95% confidence intervals or interquartile ranges.

All biomarkers of bone turnover except OPG increased in the TDF/FTC arm compared with the ABC/3TC arm (fig. 3). The difference in change from baseline was statistically significant for osteocalcin (*P* = 0.004), P1NP (*P*<0.001), CTx (*P* = 0.016), and alkaline phosphatase (*P* = 0.005) at week 48. At week 12 the changes were significant for P1NP (*P* = 0.005) and alkaline phosphatase (*P* = 0.003), but only for P1NP (*P* = 0.009) at week 4. Contrary to this, OPG increased from baseline to week 4 in the ABC/3TC arm compared with the TDF/FTC arm (*P* = 0.029); changes in OPG at week 12 and 48 were indifferent between study arms. Using linear regression we explored the association between early changes in bone turnover markers (baseline to 4 and 12 weeks, respectively) and subsequent reduction in BMD at week 48, but found that none of the markers predicted BMD loss. In addition, there were no association between baseline levels of bone turnover markers and change in BMD. As the proportion of females was slightly higher in the ABC/3TC arm we also checked for any association between gender and change in BMD but found none.

### Markers of renal dysfunction

**Figure 4:**
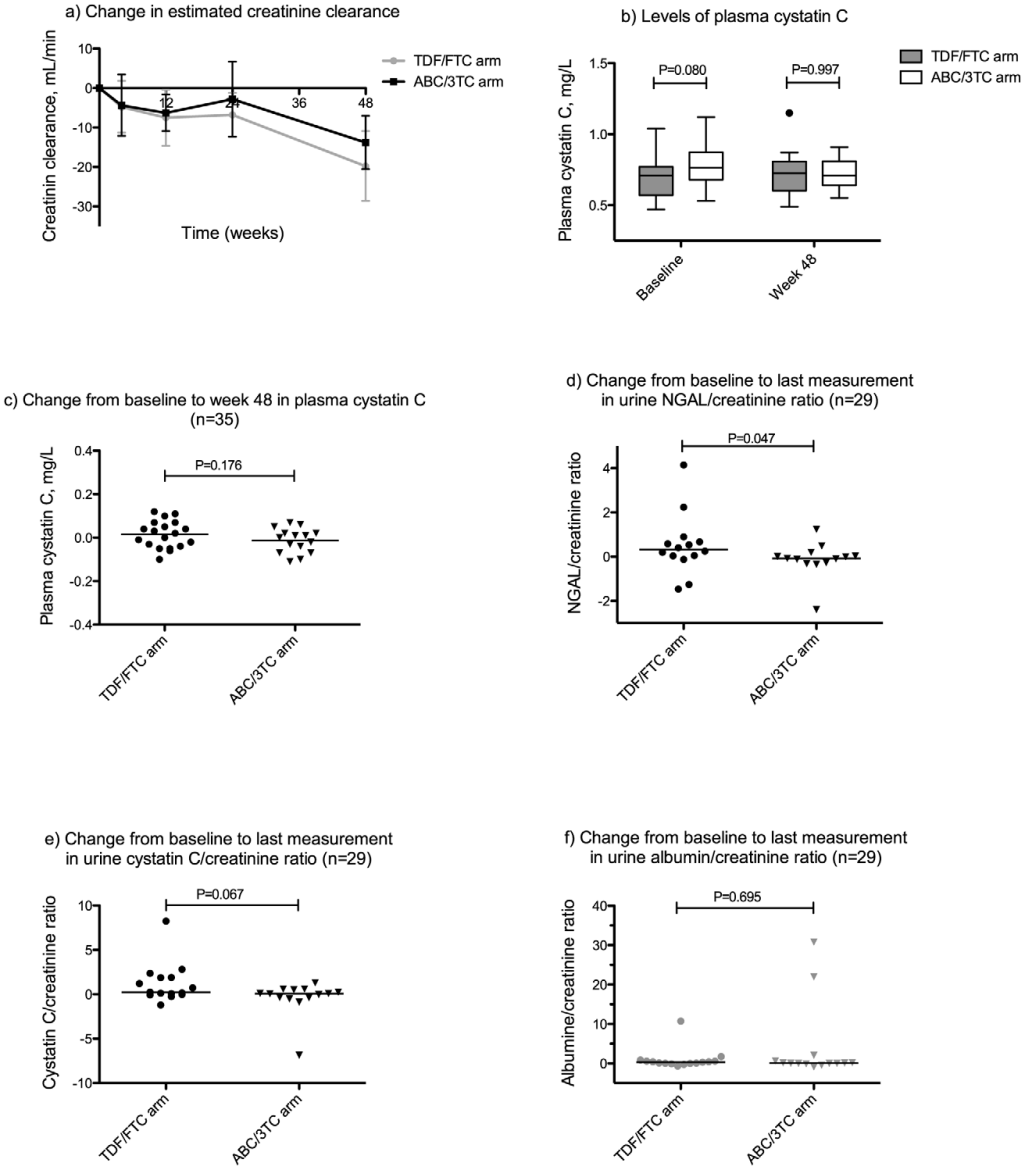
Changes from baseline in markers of impaired renal function. Changes from baseline to the indicated time points in estimated creatinine clearance (a), levels of plasma cystatin C at baseline and week 48 (b), changes from baseline to week 48 in plasma cystatin C (c), and changes from baseline to the last measurement in ratios of urinary biomarkers relative to urine creatinine concentration (d, e, f). Changes in urinary biomarkers (d, e, f) were evaluated in 29 patients (15 in the TDF/FTC arm and 14 in the ABC/3TC arm); of these, 24 were followed from baseline to week 48. Error bars in (a) show 95% confidence intervals; the horizontal line in (c) represents the mean value, while the horizontal lines in (d, e, f) represent median values. eCrCl = estimated creatinine clearance; NGAL = neutrophil gelatinase-associated lipocalin TDF/FTC = tenofovir/emtricitabine; ABC/3TC = abacavir/lamivudine.

A decrease in eCrCl from baseline was observed in both study arms that reached statistical significance at week 12, 24, and 48 in the TDF/FTC arm, and at week 12 and 48 in the ABC/3TC arm. In contrast, no significant change was observed from baseline to week 48 in the plasma levels of cysC in any of the study arms. There was no difference between study arms in the change from baseline, neither in eCrCl nor plasma cysC at any time point (fig. 4a and 4c). The changes from baseline in plasma phosphate did not differ between study arms (data not shown). Changes in urinary biomarkers were evaluated in 29 patients (15 in the TDF/FTC arm and 14 in the ABC/3TC arm); of these, 24 were followed from baseline to week 48. The urinary NGAL/creatinine ratio increased in the TDF/FTC arm compared with the ABC/3TC arm (*P* = 0.047), but the difference was marginal (fig. 4). There was a trend towards a similar increase in urinary cysC/creatinine ratio (*P* = 0.069) with TDF/FTC treatment compared with ABC/3TC, whereas the change in albumin/creatinine ratio was similar between study arms (*P* = 0.695) (fig. 4).

There was no difference in blood pressure between study arms at baseline or at the end of the study (data not shown).

## Discussion

In this randomized clinical trial among virologically suppressed HIV-infected adults, the introduction of fixed-dose treatment with TDF/FTC lead to decreases in hip and lumbar spine BMD compared with fixed-dose treatment with ABC/3TC. In addition, we detected increases in the majority of measured bone turnover biomarkers in TDF/FTC- compared with ABC/3TC-treated patients. There was no difference in renal function between the two study arms as evaluated by eCrCl or plasma cysC, but a minor increase in urinary NGAL/creatinine ratio was observed in the TDF/FTC arm compared with the ABC/3TC arm.

The strength of the study is the randomized design combined with exploratory analyses of several biomarkers of bone and renal function. Concurrent inflammation was assessed at baseline and did not differ between study groups. Furthermore, the entry criteria required that patients be negative for the HLAB5701 allele, had not previously been exposed to ABC or TDF, and had been virologically suppressed for at least 3 months prior to randomization.

Only one previous randomized clinical trial (STEAL) has evaluated BMD in virologically suppressed HIV-infected patients switching to a TDF- or ABC-based regimen [14]. In the STEAL-study TDF/FTC significantly lowered hip and spine T scores at both weeks 48 and 96, whereas both T scores increased in the ABC/3TC group. The changes in T scores in the two study arms in our study were of a similar magnitude as in the STEAL-study (data not shown). However, we used changes in BMD as the principal bone endpoint measure and found reductions in BMD with TDF/FTC-treatment, but stable rather than increased BMD with ABC/3TC-treatment. Our patient material is much smaller and, consequently, our data less robust. Biomarkers of bone turnover increased in patients treated with TDF compared with ABC in a recently presented STEAL-sub study, but early changes in these biomarkers did not predict bone loss at week 96 [30]. We measured bone turnover biomarker as early as 4 weeks after randomization, which was not done in the STEAL-sub study, but generally the changes seen in biomarkers of bone turnover in the present study are in line with the findings of the STEAL- study. In the SMART-study BMD declined more with continuous than interrupted therapy, but no consistent drug-specific association with BMD decline was found [31]. Bone turnover markers were measured in an unpublished sub-study of the SMART study and early changes (4 months after baseline) in some of these markers predicted BMD changes over 12 months [32]. However, the SMART-study was not designed to evaluate differences in BMD between patients randomized to ABC- or TDF-based treatment and is not directly comparable to our study. Two HAART-initiation randomized trials of TDF versus ABC have evaluated effects on BMD [11], [13] in which BMD reductions with TDF therapy were similar, or slightly larger, than in our material, but comparison with these are not straightforward as decreases of BMD have been observed with HAART-initiation irrespective of regimen used [7]–[10], [12].

Our results support the findings of previous studies [11], [13], [14] that TDF treatment compared with ABC is associated with a decrease in BMD, but the clinical implications are not clear. First, this should be interpreted in the context of a normal reduction in BMD after the age of 30 both in premenopausal women (∼0.3%/year), perimenopausal women (∼1%/year), and men (0–0.5%/year) [33]. Second, the loss of BMD is lesser than, but comparable to the 2–10% reduction in lumbar spine BMD that is seen with initiation of corticosteroids [34]. Still, initiation of corticosteroid therapy is associated with an increase in incident fractures [34], which has not been observed with initiation or switch to TDF-based HIV-treatment [11], [13], [14], [20], but studies so far have been too small or too short to sufficiently address this issue.

Whether the observed changes in BMD in our study and those of others are clinically relevant is a very interesting but difficult to answer question. The observed loss of BMD is quite small and not likely to confer a consequent increase in fracture risk in the majority of HIV-infected patients. At least there is no data to suggest that patients with higher risk of fractures should not receive TDF, but alternatives may be considered for those with previous fractures or known osteoporosis [35]. In addition, no increase in risk of fracture among TDF-treated patients in the STEAL-study was revealed with use of the FRAX® tool [30], but it should be noted that the FRAX® tool is not validated for patients with BMD reductions within the previous year.

It has been suggested that the initial loss of BMD may stabilize after 1 year of treatment and therefore may be caused by a continued effect of viral replication on bone turnover until complete suppression, even though other explanations have also been put forward [36]. Our data, and those of the STEAL-study, indicate a direct effect of anti-retroviral therapy (ART) on bone mass density. This is helpful in the efforts to understand how traditional risk factors for low BMD, HIV-infection and ART each contribute to the increased risk of low BMD and fractures among HIV-infected individuals.

We measured several bone turnover markers to provide additional information on the effects of these treatments on bone remodeling and potentially the mechanisms underlying the changes seen in BMD. Bone turnover markers increased in the TDF/FTC arm within the first 12 weeks after treatment switch and remained stably elevated hereafter. This indicate that loss of BMD is associated with an increased rate of bone resorption and formation that can be detected as early as 12 weeks after initiation of TDF, and possibly already after 4 weeks. The early time point evaluated in this study is particularly interesting, as comparable studies have not provided data on bone turnover biomarkers so shortly after treatment shift to a TDF-based regimen. Both OC and OPG showed initial decreases in the TDF/FTC arm followed by increase or stabilization, which raises the question if certain signaling events relevant for the bone remodeling effects of TDF are taking place within the first few weeks of treatment switch. However, this remains speculative and the meaning of these finding are uncertain. Whether the changes in markers of bone turnover are reflecting direct effects of the treatment on the osteoclasts or osteoblasts or both [37], [38] or an indirect effect via effects on the calcium homeostasis, including reduced activation of vitamin D and secondary increases in parathyroid hormone, remains to be shown [39]. It is also unclear whether these bone turnover markers can be used for monitoring patients during treatment as early changes in these markers did not predict BMD loss similarly to what was observed in the STEAL sub-study [30].

We did not detect any differences between study arms in the changes in eCrCl or plasma cysC, but still observed a minor increase in 1 of 3 measured biomarkers of renal dysfunction among patients in the TDF/FTC arm. This is comparable to the findings in the HAART-initiation ASSERT-study [22] where retinol-binding protein and β-2 microglobulin increased significantly more in the TDF/FTC group. In other HAART-initiation studies biochemical parameters of renal function were similar between TDF/FTC and ABC/3TC treated patients [19]–[21], but more patients in the TDF groups developed clinical renal impairment [19], [20]. No difference in eGFR, creatinine clearance or other renal endpoints was found among virologically suppressed HIV-patients in the STEAL-study [14].

The primary limitation of our study is the failure to reach the intended number of enrolled patients, which reduced the statistical power to detect differences between study arms. In addition, DXA was not performed in all patients and we only have 48 weeks of follow-up, which is too short to acknowledge the long-term effect on BMD reductions that appear to occur primarily within the first 24–48 weeks with stabilization hereafter. Interestingly, while eCrCl decreased over time in both study arms, no change in plasma cysC was observed indicating a stable GFR. We did not include GFR measurement by an exogenous marker and thus cannot compare these two estimates with more precise GFR-measurements. However, the eCrCl estimate based on plasma creatinine is dependent on factors other than GFR, including changes in muscle mass and tubular secretion of creatinine, and is possible that either the HIV infection or the anti-viral treatment may influence such factors. Previous studies comparing eCrCl and cysC based GFR estimates on HIV patients with normal renal function and on anti-viral treatment have generated mixed results [40], [41]. The 24-hour urine collections were not complete precluding us from determining 24-hour excretion of albumin, tubular markers, and the fractional phosphate excretion. Furthermore, due to incomplete sampling, urinary biomarkers were not measured in all patients at all time points. We did not, however, detect differences in plasma phosphate levels between study arms.

Long-term effects of HAART, including effects on bone and renal function, will continue to be outcomes of interest in future clinical trials. Beside this, more prospective studies will be needed to evaluate potential interventions to prevent or reverse loss of BMD. While several clinical trials have shown that bisphosphonate, vitamin D and calcium, either separate or in combination, are safe and effective in the treatment of decreased BMD among HIV-infected patients on HAART [42]–[45], there are still no published studies on interventions to prevent bone loss associated with TDF or HAART initiation.

In conclusion, we observed decreases in hip and lumbar spine BMD, in conjunction with early increases in bone turnover markers, in patients randomized to TDF/FTC-based treatment compared with ABC/3TC. Changes in renal function were not different between study arms although a very small increase in NGAL was detected among TDF-treated patients. The clinical significance of the latter is unclear.

## Supporting Information

Checklist S1 **CONSORT Checklist.** This manuscript is reported in accordance with the CONSORT guidelines. The Checklist S1 informs where in the manuscript the relevant information can be found.
(PDF)

Protocol S1 **Trial protocol.** English translation of the original study protocol (original language Danish).
(PDF)
