# Supplementary material for: Comparison of Bone and Renal Effects In HIV-infected Adults Switching to Abacavir or Tenofovir Based Therapy in a Randomized Trial
Source: PLoS One. 2012 Mar 29;7(3):e32445. doi: 10.1371/journal.pone.0032445 (PMC3315554; doi:10.1371/journal.pone.0032445)
Supplement: Protocol S1 — Trial protocol. English translation of the original study protocol (original language Danish). (PDF) [file pone.0032445.s002.pdf]

CLINICAL STUDY PROTOCOL

The Efficacy and Safety of Switching from Zidovudine to Tenofovir or Abacavir in HIV-infected Patients (SWAP)

An open label randomised study

STUDY NUMBER: SKS-HIV-002

STUDY NAME: SWAP-TA

VERSION DATE: July 18, 2007

1. TABLE OF CONTENTS

1. TABLE OF CONTENTS 2
2.0 INTRODUCTION AND RATIONALE 4
2.1 Side effects of antiretroviral treatment (HAART) 4
2.1.1 Lipodystrophy 4
2.1.2 Renal function 5
2.1.3 Osteopenia – osteoporosis 5
2.1.4 Hypersensitivity 5
2.1.5 Resistance 5
2.2 Hypotheses 6
3.0 Objectives 7
3.1 Primary objective 7
3.2 Secondary objectives 7
4.0 STUDY DESIGN 8
4.1 Description of the study 8
4.2 Duration of participation in the study 8
4.3 Study treatment 8
5.0 SELECTION OF PATIENTS 9
5.1 Number of patients 9
5.2. Inclusion criteria 9
5.3 Exclusion criteria 9
5.4 Inclusion period 9
6.0 TREATMENTS 10
6.1 Treatment groups 10
6.2 Concurrent treatments 10
7.0 Effect evaluation 10
7.1 Primary effect measures 10
7.2 Secondary effect measures 11
7.3 Study procedures 11
7.4. Monitoring safety 12
7.4.1. Definitions of unintended and serious unintended events 12
8.4.2 Subject withdrawal from the study 13
8.4.3 Pausing or withdrawal from the study drug 13
8.4.4 Resistance Mutations 13
8.4.5 Resistance Mutations 13
8.0 Statistical considerations 14
8.1 Sample size 14
9.0 Ethical considerations 14
10.0 MONITORING THE STUDY 15
10.1 Responsibility of the Investigator 15
10.2 Requirements on source documents 15
10.3 Use and completion of CRF records and additional requests 16
10.4 Storage of information at the centres 16
11.0 RESPONSIBILITY AND Rights of PUBLICATION 17
Principal Investigator: 17
Co-investigators: 17

Collaborators: 17
12. Amendment to protocol 18

2.0 INTRODUCTION AND RATIONALE

2.1 Side effects of antiretroviral treatment (HAART)

Combination treatment of patients with HIV has resulted in a significant improvement of survival figures. The combination therapy typically consists of a "backbone" of two reverse transcriptase inhibitors and a non-nucleoside reverse transcriptase inhibitor or a protease inhibitor.

Despite the indisputable effect of HAART on mortality and morbidity in HIV patients, a range of side effects is associated with such treatment.

A number of these arise immediately after the start of treatment and take the form of gastro-intestinal and allergic side effects. The diagnosis of these usually causes few, if any problems, and such side effects seldom cause irreversible damage.

In contrast, the use of certain types of nucleoside analogues (thymidine analogues) – such as AZT – has raised suspicions of an effect on the cellular metabolism. This may result in an inhibition of the bone marrow and a consequential reduction in the formation of CD-4 lymphocytes. This is precisely the type of cells that HIV infection eliminates. Clinical studies (Gilead 934) involving patients who had not previously received anti-HIV treatment and who commenced treatment with AZT or tenofovir (a non-thymidine analogue) have shown that the CD-4-count further increased by approx. 25% in the group treated with tenofovir (1). The effect on the cellular metabolism has also proved to result in a loss of subcutaneous fat (lipoatrophy) (2) and, possibly as part of this, to induce parts of the metabolic syndrome involving pathological glucose load and dyslipidaemia (3). These side effects seem to be triggered in particular by the deoxyribose analogues stavudine and ddI, although the most commonly utilised nucleoside analogue, AZT, also seems to have this side-effect (4). For this reason, AZT, which has been used for years as the backbone in HAART, is now being deselected in favour of an increasing use of newer nucleoside/nucleotide analogues. As a result, the nucleotide analogue tenofovir is listed as an equivalent to AZT for initial treatment in guidelines from the United States and Great Britain. In Denmark, another nucleoside analogue – abacavir – has been used to treat a number of patients who have developed symptoms of lipoatrophy. However, both abacavir and tenofovir have the potential to cause serious side effects, and experience with these substances is more limited compared with AZT.

2.1.1 Lipodystrophy

Loss of subcutaneous fat constitutes the key finding of this syndrome, while the relation to abdominal obesity is as yet uncertain. Nucleoside analogues, stavudine in particular, are associated with the syndrome, but recent studies have shown that up to 20% of patients treated with AZT develop this (5). The background for the loss of subcutaneous fat has not yet been definitively established, but the regularity and severity depend on the nucleoside analogue used and its capacity to inhibit mitochondrial polymerase γ. The inhibition of this enzyme seems to result in appreciable damage to mitochondria and subsequent apoptosis of the adipocyte (6). The extent to which this loss is irreversible is as yet unclear. A certain increase in subcutaneous fat measured through DEXA scanning has, however, been described when treatment with thymidine analogues was excanged with abacavir (7) or tenofovir (8). Similarly, in randomised clinical trials, both abacavir and tenofovir failed to produce signs of lipodystrophy during the period observed (1,9). Neither tenofovir nor abacavir show mitochondrial toxicity *in vitro* (10).

In addition to the cosmetic aspect of lipoatrophy, the condition also involves a risk of developing an impaired glucose tolerance and an atherogenic lipid profile (3).

2.1.2 Renal function

The nucleotide analogue tenofovir does not seem to result in significant lipoatrophy, and may therefore be a suitable alternative. However, in casuistic reports, the use of this substance has been associated with renal failure (11, 12). Some of these cases were observed in patients with long-term HIV infection, appreciable resistance and in combination with boosted protease inhibitors. In two major randomised studies of treatment-naïve patients, a modest reduction in renal function was initially observed, although this later stabilised and no patients developed impaired renal function (2, 13). However, a number of patients do develop hypophosphatemia, which may be associated with impaired resorption in proximal tubuli (14). Individual cases of Fanconi syndrome have also been described (15). The mechanism behind the cases described has not been definitively clarified, but it seems that damage to the proximal tubuli may play a central role. No damage by tenofovir to relevant cells has been demonstrated in tissue cultures, in contrast to other related substances such as adefovir and cidofovir (16).

2.1.3 Osteopenia – osteoporosis

Loss of bone mass (osteopenia and osteoporosis) is a normal phenomenon in people with advanced age. Certain pathological conditions such as malabsorption, primary hypogonadism, renal insufficiency and treatment with corticosteroids accelerate this process. HIV infection or treatment of HIV similarly seems to result in pathological bone loss. Thus 33–58% of the HIV patients examined had osteopenia identified by DEXA scanning in three cross-section examinations (17, 18, 19). Treatment with protease inhibitors seemed to induce osteopenia in one of these studies (17), but this could not be confirmed in other studies (18, 20). The underlying mechanism is unclear, but the effect of pro-inflammatory cytokines (TNF-α and IL-6) have been postulated as one of several possible causes (21).

In a randomised study of treatment-naïve patients, a significantly greater loss of bone mass of lumbar vertebrae was observed in the group of patients treated with tenofovir as compared with stavudine treated patients than in those treated with stavudine. This difference was not significant when the upper part of the femur was examined. The reason for these differences is not known.

2.1.4 Hypersensitivity

Abacavir produces a hypersensitive reaction in approximately 3–5% of all patients (22). The reaction is linked to the presence of the tissue type HLA B5701 (23). The symptoms are usually fever and a rash, but may be difficult to differentiate from pulmonary or gastrointestinal infections. Life-threatening complications have been observed following the recommencement of treatment after a pause (24).

No similar reactions have been described with the other nucleoside/nucleotide analogues.

2.1.5 Resistance

Resistance to antiviral drugs developing during a specific regimen followed by full or partial viral suppression by other antiretroviral drugs will lead to the development of quasispecies carrying resistance mutations. These minority species cannot be detected with the method that is usually

applied the threshold of detection being around 20% of the existing mutations, and a relatively high viral load (approx. 1,000 copies/ml) is required before the method can be used.
It is, of course, important to be able to identify such mutations in connection with the choice of subsequent treatment regimes.
With the HIV-SNaPshot method, it is possible to examine samples with a viral load as low as 20 copies/ml and to detect mutations occurring with a frequency of 5 to 10%. For technical reasons the method is applicable only on subtype B virus.
When switching treatment from AZT to either abacavir or tenofovir, the presence of a range of mutations will be of significance to the result of treatment.
In the present project the presence of the following mutations, if present, will be looked for: TAM mutations M41L and T215Y/F, which is important to the HIV virus sensitivity to tenofovir, K70R, L210W and the multi-drug resistance mutation Q151M, which constitute important resistance to AZT and, in greater numbers, to abacavir. Other important mutations are M184V and K65R, which are observed in patients being treated with abacavir and tenofovir, and whose development is inhibited by TAM mutations.
For patients in simultaneous treatment with the non-nucleoside analogues efavirenz or viramune, the K103N, Y181C and G190A/E mutations are important.

2.2 Hypotheses

1) Signs of affected renal function are observed in patients who switch to tenofovir as compared to patients treated with AZT and abacavir.
2) Loss of bone mass is observed in patients treated with tenofovir compared to patients treated with AZT and abacavir.
3) A switch from AZT to another nucleoside/nucleotide analogue (tenofovir or abacavir) increases the number of CD4 positive lymphocytes.
4) AZT causes/exacerbates lipoatrophy.
5) Tenofovir and abacavir do not cause *de novo* lipodystrophy, and patients suffering from lipodystrophy who switch to treatment with these drugs do not experience a worsening of the condition.
6) There is no deterioration of the virological control following a switch from AZT to abacavir or tenofovir.

We plan to clarify the hypotheses listed above by comparing a prior treatment regime involving AZT to one that involves abacavir or tenofovir.

3.0 Objectives

3.1 Primary objective

- To assess the profile of side effects among HIV-infected patients switching from AZT to either abacavir or tenofovir with regard to the following:
    - To compare the change in renal function (including tubule function) as assessed by specific blood- and urine analyses in the two groups (switching to abacavir or tenofovir) at week 4, 8, 12, 24, 48, and 96
      NB: tubule function not assessed at week 4 and 8.
    - To compare the change in bone mass density and bone metabolism as assessed by DEXA scan and specific blood analyses in the two groups (switching to abacavir or tenofovir) at week 4, 8, 12, 24, 48, and 96
      NB: DEXA not performed at week 12
    - To compare the change in lipoatrophy as assessed by patient questionnaire, medical examination and DEXA scan in the two groups (switching to abacavir or tenofovir) at week 4, 8, 12, 24, 48, and 96
      NB: DEXA not performed at week 12
    - To compare the change in insulin sensitivity as assessed by HOMA value in the two groups (switching to abacavir or tenofovir) at week 4, 8, 12, 24, 48, and 96

3.2 Secondary objectives

- To assess the change in CD4+ T-cell count and CD4+ T-cell subsets from baseline to week 96 in the two groups (switching to abacavir or tenofovir)

- To compare the antiviral efficacy in the two groups (switching to abacavir or tenofovir) at week 4, 8, 12, 24, 48, and 96 defined as the fraction of patients with viral load below the level of detection (<40 copies per ml)

- To compare the lipid profiles as assessed by fasting triglycerides, HDL and LDL cholesterol in the two groups (switching to abacavir or tenofovir) at week 12, 24, 48, and 96.

- To evaluate whether the presence of specific resistance mutations (established through the HIV-SNaPshot method) can predict the treatment response evaluated through rise in the number of CD positive lymphocytes and viral load at week 48.

- To establish whether specific resistance mutations (evaluated through HIV-SNaPshot) are induced differently within the two treatment groups.

- To evaluate the frequency of adverse events and severe adverse events (including hypersensitivity reactions) from baseline to week 48.

- To evaluate the scope of the change in treatment after randomisation as a result of adverse events.

4.0 STUDY DESIGN

4.1 Description of the study

The study will be an open label randomised, 2-arm study, in which patients suffering from HIV will be randomized to switch from AZT to tenofovir or abacavir. The other anti-HIV medication will be continued. The patients will be stratified after protease inhibitor use and randomised equally in a ratio of 1:1 at time 0.

The patients will be recruited from the patient clientele linked to the department of infectious medicine, department Q, at Skejby Hospital. After an eligibility assessment based on inclusion and exclusion criteria, the patient population will be randomised by drawing lots (the envelopes will contain information about the subsequent treatment).

Control visits to the outpatients' department will be carried out in weeks 4, 8, 12, 24, 48 and 96 after the baseline visit at which the randomisation took place. After the 96 weeks, patients will have the option of further follow-up on secondary effect measures up until week 144.

4.2 Duration of participation in the study

The duration of the study as regards primary effect measurements will be 96 weeks. After this patients will be followed every 16 weeks as part of routine medical treatment in line with the department's standard guidelines for the control of HIV patients in treatment. It will be possible to perform data collection regarding side effects up until week 144 after switching treatment.

4.3 Study treatment

Tenofovir is the active drug in Viread® and also part Truvada® together with emtricitabin. Both drugs are manufactured by Gilead and distributed in Denmark by Swedish Orphan.

Viread®: 245 mg tenofovirdisoproxil, 1 tablet daily
Truvada®: 245 mg tenofovirdisoproxil and 200 mg emtricitabin, 1 tablet daily

Abacavir is the active drug in Ziagen® and also part of Kivexa® together with lamivudine. Both drugs are manufactured by GlaxoSmithKline Pharma A/S.

Ziagen®: 300 mg, 2 tablets once daily
Kivexa®: 600 mg abacavir and 300 mg lamivudine, 1 tablet daily

The study drugs will be labelled as investigational drug and delivered by Aarhus University Hospital Pharmacy. The study subjects will receive the investigational drugs free of charge (as is standard practice). Remaining pills will be counted when study subjects receive new medicine every 3 months and the result will be listed. In addition, all delivered, used and returned medicine will be accounted for.

5.0 SELECTION OF PATIENTS

5.1 Number of patients

It is planned to include 45 patients in each of the two groups – i.e. a total of 90. This number will allow comparison of the regimes involving tenofovir and abacavir.

5.2. Inclusion criteria

- HIV patients with a viral load below the level of detection (<40 copies/ml) and a treatment regime that includes AZT for a minimum of three months.
- Age >=18 years
- If woman of child bearing potential: negative pregnancy test
- If woman of child bearing potential: safe anti-conception
- Negative HBsAg test within 3 months of inclusion

5.3 Exclusion criteria

- Previously treatment with abacavir or tenofovir
- Previous genotypic resistance examination compatible with resistance to abacavir or tenofovir
- Tissue type HLA-B5701
- Known renal disease
- Untreated hypertension
- Known diabetes mellitus
- Known osteoporosis
- Pregnant or nursing women
- Fertile women who do not use safe contraception or practice complete abstinence from sex
- Patients with illegal drug habits
- Alcohol abuse that complicates the ability to follow the treatment regime or the protocol evaluation
- Patients with ALT > 5 x the upper normal value.
- Female patients must not be pregnant at the start of the study. This means they have to present a negative pregnancy test at the start of the study.

5.4 Inclusion period

Subjects can be included in the study from September 1, 2007 to August 31, 2008.

6.0 TREATMENTS

6.1 Treatment groups

Please, refer to 4.3 (Study treatment) for description of the investigational drugs.

Group 2 (the tenofovir group). The patients in this group are switched from AZT to tenofovir disoproxil, 245 mg x 1 daily, and continue to take their other antiviral medication unchanged.

Group 3 (the abacavir group). The patients in this group are switched from AZT to abacavir, 600 mg x 1 daily, and continue to take their other antiviral medication unchanged.

All the medications have been registered in Denmark for HIV treatment.

6.2 Concurrent treatments

All other medicine is registered in the CRF.

7.0 Effect evaluation

7.1 Primary effect measures

- Kidney function assessed by Cystatin C baseline and at 4, 8, 12, 24, 48, and weeks after. Kidney function assessed by creatinine clearance at baseline and at 12, 24, 48, and 96 weeks.
- Blood and urine levels of markers of tubule function at baseline and at 12, 24, 48, and 96 weeks.
- Bone mass density as assessed by DEXA scan at baseline and at 24, 48, and 96 weeks
- Blood and urine levels of bone turnover markers at baseline and at 12, 24, 48, and 96 weeks
- Insulin sensitivity at baseline and at 12, 24, 48, and 96 weeks
- Change in body composition established by the physician and the patient through a questionnaire and a standardised examination at baseline and at 12, 24, 48, and 96 weeks
- Change in the amount of subcutaneous fat established through DEXA scanning at baseline and after 24, 48 and 96 weeks.

7.2 Secondary effect measures

- The fraction of patients with <40 HIV-RNA copies 4, 8, 12 and 24, 48 and 96 weeks after the start of treatment.
- CD4+ t-cell count and the distribution of T-cell subsets as assessed through routine flow cytometry at the Department of Clinical Immunology, Skejby Hospital, at the planned controls at week 0 (baseline) and weeks 4, 8, 16, 24 and 48.
- Change in solid triglyceride, HDL and LDL cholesterol at baseline and after 4, 8, 12, 24, 48 and 96 weeks.
- Incidence of resistance mutations evaluated through the HIV-SNaPshot at week 0 (baseline) and at weeks 4, 8, 12, 24 ,48 and 96
- The number of patients experiencing adverse events during the study period (subdivided by the organ affected, relation to the study medicine, and by level of severity).
- The number of patients experiencing serious adverse events during the study period (subdivided by the organ affected, relation to the study medicine, and by level of severity).

7.3 Study procedures

Follow-up and clinical control will be according to the below outlined schedule:

	Baseline	Week 4	Week 8	Week 12	Week 24	Week 48	Week 96
Clinical control incl. accounting for medicine and adverse event	X	X	X	X	X	X	X
Routine biochemistry[1] and HIV-RNA, resistance and CD4+ T-cell count[2]	X	X	X	X	X	X	X
Cystatin C[3]	X	X	X	X	X	X	X
Bone turnover markers (blood and urine)[4]	X			X	X	X	X
Markers of renal tubular function (blood and urine)[5]	X			X	X	X	X
Fasting lipids	X			X	X	X	X
Fasting glucose and serum insulin for HOMA-value[6]	X			X	X	X	X
DEXA	X				X	X	X
Lipoatrophy questionnaire[7]	X			X	X	X	X
Anthropometrics and BP	X			X	X	X	X

1) The routine analyses comprise: Hb, leukocyte and differential counts, thrombocyte count, S-creatinine, S-sodium, S-potassium, ALAT, bilirubin, alkaline phosphatase, coagulation factors II+VII + X, amylase. Department of Clinical Biochemistry, Aarhus University Hospital, Skejby.
2) HIV-RNA/viral load and CD4+ T-cell count will be performed at the Department of Clinical Microbiology, Aarhus University Hospital, Skejby.
3) Cystatin C will be measured at Department of Nephrology, Aarhus University Hospital, Skejby.
4) Blood: PTH, 25-hydroxyvitamin D, 1,25 dihydroxyvitamin D, Fibroblastic Growth Factor 23 (FGF23), frizzled-related protein, Phosphate, aminoacids (all aminoacids by HPLC) Urine: Potassium, Sodium, Bicarbonate, Phosphate, Creatinin, cyclic-AMP, aminioacids (all aminoacids by HPLC), pH.
5) Blood: PTH, 25-hydroxyvitamin D, 1,25 dihydroxyvitamin D, osteocalcin, alcaline phosphatase and bone-specific alcaline phosphatase, C-terminal telopeptide of type 1 collagen. Urine: Morning urine (2nd void): Cross-linked terminal telopeptide of collagen type 1.
6) Fasting glucose and insulin will be measured at the Department of Infectious Diseases, Aarhus University Hospital, Skejby and used for determining HOMA-value
7) Patient questionnaire that has been used in previous studies as a subjective assessment of changes in the fat distribution.

7.4. **Monitoring safety**

7.4.1. **Definitions of unintended and serious unintended events**

An adverse event (AE) refers to any unwanted medical condition in a patient who has received a medical product, even though there may be no causal link with the medical product.
A priori, an effect end point will not be considered an unintended event unless the progress, severity or other conditions concerning such an event are considered by the Investigator (according to his/her best medical evaluation) to be exceptional in relation to the medical condition of the patient.

An serious adverse event (SAE) refers to any unintended event which, irrespective of the dose:

- results in death, or
- is life-threatening, or
- requires hospitalisation or extension of current hospitalisation, or
- results in chronic or significant invalidity or incapacity to work, or
- is a congenital anomaly/malformation, or
- is a significant clinical event:

Unintended events will be registered in the CRF.

8.4.2 Subject withdrawal from the study

Study subjects may withdraw from the study due to the following reasons:

1. Protocol violation
2. Any adverse event, where - in the opinion of the investigator – continued participation in the study will result in an unacceptable risk to the patient
3. Increase in s-creatinin >50% compared to baseline in two consecutive measurements separated with one week
4. Suspicion of allergic reaction toward the study drugs
5. Patient request

Withdrawn subjects will not be replaced.

8.4.3 Pausing or withdrawal from the study drug

Study subjects should pause their medication in the following situations:

1. Increase >5 x baseline ALAT eller >10 x ULN in two consecutive measurements separated with one week

   Treatment may be resumed when ALAT < 2 x baseline. S-ALAT should hereafter be monitored. Suspicion of hepatic dysfunction caused by other factors than the study drug must be addressed medically. In the case of renewed increases in ALAT meeting the criteria described in 1) the study subject must be withdrawn from the study.

2. If judged necessary by the co-investigator for medical reasons

Study subjects that withdraw from the study drug will be asked to complete follow-up.

8.4.4 Resistance Mutations

If a study subjects display signs of resistance defined as viral load >400/ml the test must be repeated after minimum 4 weeks. In the case of viral load still above 400/ml there should be performed resistance analyses at Statens Serum Institut and depending on the result withdrwal from the study drug may be necessary.

8.4.5 Resistance Mutations

All adverse events are recorded in the CRF. All suspected unexpected serious adverse reactions (SUSAR) must be reported to Sponsor-Investigator by fax. Sponsor-investigator must report SUSAR to the Danish Medicines Agency within 7 days (lethal or life threatening) / 15 days (all others). AE and SAE will be reported annually to the Danish Medicines Agency and the research ethics committee according to current guidelines. All AE and SAE must be reported to the Danish Medicines Agency in a final report upon completion of the study.

8.0 Statistical considerations

8.1 Sample size

There is limited knowledge on the frequency of renal impairment, including tubular function with tenofovir or abacavir treatment. In a study by Kinai et al 71% of the patients were found to develop impaired tubular function within 12 weeks as assessed by B2-microglobuline excretion. Exact figures regarding abacavir is not known, but it is assumed that tubular function is affected in 50% of treated patients. Using this assumption, a test power of 80%, a 5% significance level and expecting a 10% drop-out rate, 90 participants are needed in the study. Regarding all other biomarkers to be measured in this study there is currently no knowledge to allow a valid power calculation and the assessment of these markers must be considered exploratory.

The primary effect measurements will be calculated on the basis of the "per-protocol" population. This means all patients that had the analysis in question performed. Safety assessment will be evaluated on all patients receiving at least one dose of the study drugs.
For continuous variables the measurements will be analyzed by either Student's T-test or a corresponding non-parametric test (in the event that normal distribution is not observed).
Categorical variables will be evaluated using either a Chi^2-test or Fisher's exact test. Safety data will be presented in table form with a statement of organ affected, relation to the study medicine and degree of severity.
Treatment compliance will be presented with statements of the mean value, standard deviations, median, minimum and maximum values. In addition, the proportion of patients who are <80% compliant will be presented for each treatment group. The number of patients for whom theoretical numbers of tablets were used in the calculation of compliance will be presented for each treatment group.

9.0 Ethical considerations

This clinical study will be carried out in accordance with the principles laid down at the "18th World Medical Assembly (Helsinki, 1964)", all applicable supplements laid down by "World Medical Assemblies", and the "ICH guidelines for Good Clinical Practice" (GCP).
The study is to be approved by the regional ethical committee(s) before it is initiated.
The three treatment regimes are estimated to represent the same antiretroviral strength. The study is intended to clarify whether there are differences between the side effects profiles of the two treatments with regard to type and severity of side effects.
Both study drugs are registered, and the risk of serious side effects is considered small. Moreover, all the patients will be evaluated closely during the project. The treatments applied are already used today without the monitoring for the side effects that can be offered by the study.
The knowledge that can be drawn from studies is considered to outweigh the risk of discomfort and risks to the patient. The study may well help to define which of the two treatments should be preferred as the nucleoside analogue backbone in the future treatment of HIV.
DEXA full-body scanning applies a radiation dose of <5 μSv (background radiation 4 μSv/year).
The data will be anonymised no later than at the end of the project. Patients will be entitled to ask to be informed of their own examination results.

The study will be discontinued in case one or both of the study treatments imposes an unacceptable health risk to the patients.

Information for patients
Patients will be informed about the project in connection with the planned control visits; here, an appointment for an information meeting will be agreed upon. This meeting will be held in an undisturbed room, and patients will be given the pamphlet entitled "Before you decide"

10.0 MONITORING THE STUDY

10.1 Responsibility of the Investigator

The Investigator will be entitled to appoint such Sub-Investigators as he/she may consider appropriate. These people will then be able to assist the Investigator in the performance of the study pursuant to the clinical study protocol. All Sub-Investigators are to be appointed and their names entered on a list well in advance of initiation of the study-related procedures. The Sub-Investigators are to be supervised by the Investigator, under whose responsibility they will also be. The Investigator will supply them with a study protocol and all the information necessary.

The Investigator of this clinical study is likewise responsible to the health care authorities for taking all reasonable steps to ensure correct performance of the study with regard to ethics, and observance of the integrity and validity of the data collected and registered in the CRF. Thus the primary function of the Monitoring Team is to assist the Investigator in maintaining a high level of ethics, scientific character, as well as technical and legislation-related quality in all aspects of the clinical trial.

At regular intervals, the centre will be contacted – through monitoring visits, letters or phone calls – by a representative of the Monitoring Team with a view to evaluating the progress of the project. The compliance of the Investigator and the patients in relation to the protocol and to any problems that may have arisen will similarly be monitored. During these monitoring visit, the Monitoring Team will discuss with the Investigator points including, but not limited to, the following: informed consent of the patients, patient recruiting and follow-up, documentation and reporting of serious unintended events, allocation of study medicine, patient compliance with the treatment regime for the study medicine, simultaneous treatment and data quality.

Sponsor-Investigator will no later than 90 days after study completion report this to the Danish Medicines Agency, including a report of the findings of the study.
The study is reported to the Danish Data Protection Agency and the International Committee of Medical Journal Editors (ICMJE) initiative at http://prsinfo.clinicaltrials.gov

Both positive and negative results from this trial will be published.

10.2 Requirements on source documents

The following are regarded source documents:

1. Labka print-outs
2. Written or printed results from any biochemical, immunological, virological or radiological analysis performed in the study.

The following will be noted directly in the CRF:

1. Answers from the lipoatrophy questionnaire
2. Anthropometric data and BP
3. List of medical therapy
4. Medical conditions and adverse events list
5. List of HIV-associated diseases
6. Viral load and CD4+ T-cell count results

10.3 Use and completion of CRF records and additional requests

The Investigator is responsible for keeping precise and updated CRF records with the purpose of registering all observations and other data that concern this clinical study. All CRF records are to be completed in full with a level of care and attention sufficient to ensure precise interpretation of the data.

If it becomes necessary to make a correction, the previous information must not be overwritten. The corrected information is to be entered next to the original information, and the entry is then to be dated and signed.

Collection of CRF records will normally be carried out during each visit. Registrations concerning simultaneous medication will be made during each visit.

10.4 Storage of information at the centres

The Investigator is to store all study documentation in a confidential manner and take steps to prevent incorrect or untimely destruction of these documents.
The informed consent statement will contain a statement to the effect that the patient allows the Sponsor's authorised staff, the scientific ethical committee system and health care authorities to have direct access to source data that support data in the CRF (the patient's journal, appointment books, original laboratory responses, etc.). The staff, which is bound by a confidentiality obligation, is to keep all information about personal identity and/or personal medical information confidential (pursuant to confidentiality regulations).
It is recommended that the Investigator store the study documents for at least fifteen (15) years following the conclusion or termination of this clinical study.
However, any legislative requirements that stipulate a longer period of storage should be taken into account.
If, as a result of his/her personal situation, the Investigator is no longer able to guarantee appropriate storage of the documents, the Investigator is to inform the Sponsor of this, and the relevant documents are then to be transferred to a different, mutually agreed storage location.

11.0 RESPONSIBILITY AND Rights of PUBLICATION

Principal Investigator:

Consultant Alex Lund Laursen, MD – Dept. Q, Aarhus University, Skejby

Co-investigators:

Senior Consultant Lars Østergaard, MD – Dept. Q, Aarhus University, Skejby

Consultant Carsten Schade Larsen, MD – Dept. Q, Aarhus University, Skejby

Collaborators:

Study monitor: The GCP Unit, contact person Birgitte Schlemmer

The Arhus University Hospital Pharmacy, contact person: Karen Sillesen

Department of Nephrology, Aarus University Hospital, Skejby, contact: Per Ivarsen

Department of Endocrinology, Aarus University Hospital, contact: Bente Langdahl

Department of Clinical Biochemistry, Aarus University Hospital, Skejby, contact: Jan Møller

Department of Infectious Diseases, Research Unit, Aarhus University Hospital, Skejby, contact: Martin Roelsgaard Jakobsen

12. Amendment to protocol

The objective of the SWAP study that has been approved by the research ethics committee is to evaluate and compare the safety profile of two of the most frequently used drugs in the treatment of HIV-infection: abacavir and tenofovir. These two drugs is now widely preferred to zidovudine as stated in the primary study protocol. Until now abacavir and tenofovir have been regarded as equal with regard to cardiovascular safety.

However, at the latest Conference on Retroviruses and Opportunistic Infections CROI) data from a large cohort study was presented that suggested an increased cardiovascular risk with abacavir treatment. These are preliminary data and have not caused any changes in the treatment guidelines, but it is of importance to evaluate the risk in randomized trials. The data did not imply a cumulative effect, so if an increased risk were present this would likely be caused by inflammatory or coagulatory mechanisms. It would therefore be important to include an assessment of these mechanisms in the coming SWAP study.

We therefore ask to include the following analyses in the study:

ICAM, VCAM, E-Selectin, hs-CRP, IL-6, D-dimer, and P-fibrinogen

These biomarkers reflect inflammation and coagulation and the results from the study may contribute to clarify the relative risk with use of these drugs. These analyses or results are judged not to burden the study participants as they closely related to the objectives already stated in the protocol.

References

1 Gallant JF, De-Jesus F, Arribas JR et al. Rofovir DF, emticitabine, and efavirenz vs zidovudine, lamivudine, and efavirenz for HIV. NEJM 2006;354 :251-60.

2 Brinkman K, ter Hofstede HJ, Burger DM et al. Adverse effects of reverse transcriptase inhibitors: mitochondrial toxicity as common pathway. AIDS 1998;12:1735-44.

3 Grunfeld C and Tien P. Difficulties in understanding the metabolic complications of acquired immune deficiency syndrome. Clin Infect Dis 2003; 37 suppl 2:43-6.

4 Joly V, Flandre P. Meiffredy V et al. Increased risk of lipoatrophy under stavudine in HIV-1 infected patients: results from a comparative trial. AIDS 2002;16:2447-54.

5 Nolan D, Hammond E, James I, McKinnon E, Mallal S. Contribution of nucleoside-analogue reverse transcriptase inhibitor therapy to lipoatrophy from the population to the cellular level. Antivir Ther 2003;8:617-26.

6 McComsey GA and Walker VA. Role of mitochondria in HIV lipoatrophy: insight into pathogenesis and potential therapies. Mitochondrion 2004;4:111-8.

7 Moyle GJ, Baldwin C, Langroudi B. A 48-week randomized, open-label comparison of three abacavir-based substitution approaches in the management of dyslipidemia and peripheral lipoatrophy. JAIDS 2003;33:22-8.

8 Moyle G, Sabin C, Cartledge J et al. Arandomized, open-label comparative study of tenofovir DF vs. abacavir as substitute for a thymidine analogue in persons with lipoatrophy and sustained virological suppression on HAART. 12th Conference on Retrovirus and Opportunistic Infections. 22-25 February 2005. Boston, MA.,U.S.A.

9 Shlay JC, Visnegarwala F, Bartsch G, Wang J et al. Body composition and methabolic changes in antiretroviral – naïve patients randomized to didanosine and stavudine vs. abacavir and lamivudine. JAIDS 2005;38:147-55

10 Birkus G, Hitchcock MJM, Ciklar T. Assessment of mitochondrial toxicity in human cells treated with tenofovir: comparison with other nucleoside reverse transcriptase inhibitors. Antimicrob Ag Chemother 2002;40:716-23.

11 Peyriére H, Reynes J, Rouanet I et al. Renal tubular dysfunction associated with tenofovir therapy: report of 7 cases. JAIDS 2004;35:269-73.

12 Coca S, Perazella MA. Acute renal failure associated with tenofovir: evidence of drug-induced nephrotoxicity. Am J Med Sci 2002;324:342-4.

13 Gallant JE, Staszewski S, Pozniak AL et al. Efficacy and safety of tenofovir DF vs. stavudine in combination therapy in antiretroviral-naïve patients: a randomized trial. JAMA 2004;292:191-201.

14 Badiou S, Merle de Boever C, Terrier N et al. Is tenofovir involved in hypophosphatemia and decrease of tubular phosphate reabsorption in HIV-positive adults? J Infect 2006;52(5)335-8.

15 Karras A, Lafaurie M, Furco A et al. Tenofovir-related nephrotoxicity in human immunodeficiency virus-infected patients: three cases of renal failure, Fanconi syndrome, and nephrogenic diabetes isipidus. Clin Infect Dis 2003;36:1070-3

16 Cihlar T, Birkus G, Greenwalt DF et al. Tenofovir exhibits low cytotoxicity in various human cell types: comparison with other nucleosode reverse transcriptase inhibitors. Antivir Res 2002;54(1):37-45.

17 Tebas P, Powderly WG, Claxton S et al. Accelerated bone and mineral loss in HIV-infected patients receiving potent antiretroviral therapy. AIDS 2000;14:63-7.

18 Carr A, Miller J, Eismann JA et al. Osteopenia in HIV-infected men: association with asymptomatic lactic academia and lower weight pre-antiretroviral therapy. AIDS 15:703-9.

19 Faifield WP, Finkelstein JS, Klibansky A et al. Osteopenia in eugondal men with acquired immune deficiency syndromewasting syndrome. J Clin Endocrinol Metab 2001;86:2020-6.

20 Nolan D, Upton R, McKinnon E et al. Stable or increasing bone mineral density in HIV-infected patients treated with nelfinavir or indinavir. AIDS 2001;15:1275-80.

21 Thomas J and Doherty SM. HIV infection – A risk factor for osteoporosis. JAIDS 2003;33:281-91.

22 Hewitt R. Abacavir hypersensitivity reaction. Clin Infect Dis 2002;34:1137-42

23 Mortio A, Nolan D, Almeida CA. Predicting and diagnosing abacavir and nevirapine drug hypersensitivity from bedside to bench and back again. Pharmacogenomics 2006;7:15-23.

24 Walensky RP, Goldberg JH, Daily JP. Anaphylaxis after rechallenge with abacavir. AIDS 1999;13:999-1000.
